# Gene Model Related to m6A Predicts the Prognostic Effect of Immune Infiltration on Head and Neck Squamous Cell Carcinoma

**DOI:** 10.1155/2021/1814266

**Published:** 2021-08-20

**Authors:** Yaping Deng, Kehua Li, Fengwu Tan, Hanbo Liu

**Affiliations:** Department of Otolaryngology Head and Neck surgery, The Affiliated Zhuzhou Hospital Xiangya Medical College CSU, ZhuZhou, Hunan, China

## Abstract

Head and neck squamous cell carcinoma (HNSCC) is a highly aggressive solid tumor. Because most studies have focused on the intrinsic carcinogenic pathways of tumors, we focused on the relationship between N6-methyladenosine (m6A) and the prognosis of HNSCC in the tumor immune microenvironment. We downloaded RNA-seq data from the TCGA dataset and used univariate Cox regression to screen m6A-related lncRNAs. The expression value of LASSO-screened genes was the sum of LASSO regression coefficients. We then evaluated relationships between the risk score and cellular components or cellular immune response. Differences in immune response under various algorithms were visualized with heat maps. The GSVA package in R was used to analyze GO, BP, KEGG, and hallmark gene sets of immune checkpoint clusters and immune checkpoint scores. The GSEA analysis was performed with the cluster profile package, yielding 21 m6A genes. Related lncRNAs were screened with Pearson's correlations, and the resulting 442 lncRNAs were screened using single-factor analysis. Eight lncRNAs closely related to prognosis were identified through survival random forest. Survival analysis showed that patients with a high risk score had a poor prognosis. Low- and high-risk-score groups differed significantly in m6A gene expression. Prognostic scores from different algorithms were significantly correlated with B cells, T cells, and memory cells in the immune microenvironment. Expression of immune checkpoints and signal pathways differed significantly across risk-score groups, suggesting that m6A could mediate lncRNA-induced immune system dysfunction and affect HNSCC development. A comprehensive study of tumor-cell immune characteristics should provide more insight into the complex immune microenvironment, thus contributing to the development of new immunotherapeutic agents.

## 1. Introduction

Head and neck squamous cell carcinoma (HNSCC) is the sixth most common malignant tumor worldwide, claiming approximately 350000 lives every year [1]. HNSCC includes malignant tumors of the oral cavity, nasopharynx, oropharynx, hypopharynx, larynx, nasal cavity, and salivary gland [2]. Patterns of clinical behavior and treatment response to recurrent and metastatic HNSCC are heterogeneous. Available treatment strategies range from potential salvage surgery and reirradiation to palliative systemic therapy and optimal supportive treatment [3]. The emergence of new treatment options may improve disease control and prolong survival after surgical methods and techniques with greater complexity, such as highly conformal and accurate radiation techniques, as well as immunotherapy [4].

For many reasons, HNSCC is sometimes difficult to treat. These include the effects of previous treatments on tumor cells and the infiltrative and multifocal nature of HNSCC, which are typical features of recurrent disease in this region [5]. A review has shown that HNSCC is suitable for immunotherapy. Immune escape plays a key role in the occurrence and development of tumors [6]. Here, we analyzed the relationship between genetic models related to immune infiltration and biometrics-based prognosis of HNSCC.

Increasing evidence suggests that long noncoding RNAs (lncRNAs) are related to human diseases. Next-generation sequencing has identified tens of thousands of lncRNAs in numerous organisms, ranging from single-celled eukaryotes to humans [7]. Because of their tissue- and cell-type-specific expression, lncRNAs are potential cancer biomarkers [8]. They have complex and extensive HNSCC development functions, including cancer growth, recurrence, and metastasis [9]. Their expression patterns in HNSCC are irregular and specific [10]. Although the relationship between lncRNAs and HNSCCs remains unclear, some lncRNAs are abnormal and contribute to the cancer's occurrence and development [11, 12]. Enhancer RNA (eRNA) is a subclass of lncRNAs transcribed in gene enhancers, the main cis-regulatory elements in the genome [13]. Transcriptional regulation of eRNAs plays a role in cancer [14]. N6-methyladenosine (m6A), which was first discovered in the 1970s, is recognized as the most prominent and abundant form of internal modification that occurs in messenger RNAs (mRNAs) and long noncoding RNAs (lncRNAs) in many eukaryotic species [15, 16]. m6A methylation is thought to affect every aspect of RNA metabolism, including RNA splicing, translocation, stability, and translation into protein [17]. Here, we aimed to identify prognostic eRNAs and their target genes in HNSCC.

Immunotherapy activates the host's natural defense system, which then recognizes and eliminates tumor cells. It is an effective treatment method with unparalleled synergistic survival advantages in a variety of cancers [18]. The development of tumor gene-expression profiles enabled identification of prognostic expression characteristics and patient selection for targeted therapy. Recent studies have evaluated correlations of immune-related gene expression in patients whose various solid tumors were treated with immunotherapy [19]. Tumor-infiltrating immune cells play a vital role in tumor spread, recurrence, metastasis, and treatment response to immunotherapy [20, 21]. For example, tumor-associated macrophages (TAMs) secrete immunosuppressive cytokines (e.g., interleukin 10 (IL-10) and transforming growth factor-*β* (TGF-*β*)) and inhibit host antitumor activity, thereby promoting tumor progression [22]. In contrast, increased levels of tumor-infiltrating lymphocytes (TLS), such as CD4+ T cells and CD8+ T cells, are associated with elevated survival rates and tumor responsiveness [23]. The activation of T cells and immune checkpoint molecules is essential for anticancer immune response [24]. In this study, we performed immune infiltration analysis of the prognostic model. We analyzed the expression of immune checkpoints in individuals with high- and low-risk scores.

The clinicopathological data we collected included sex, age, stage, grade, survival status, and survival duration. A prognosis score was developed and verified. The m6A-based prognosis model and its clinical characteristics were analyzed. We then investigated immune infiltration, as well as the expression of immune checkpoints and signaling pathways, in different risk-score groups. A comprehensive study on immune cells, immune-related factors, cytokines, and immune characteristics of tumor cells during HNSCC may provide more insight into the complex immune microenvironment, thereby contributing to the development of new immunotherapeutics.

## 2. Results

### 2.1. Establishment and Validation of Prognostic Score

**Figure 1:**
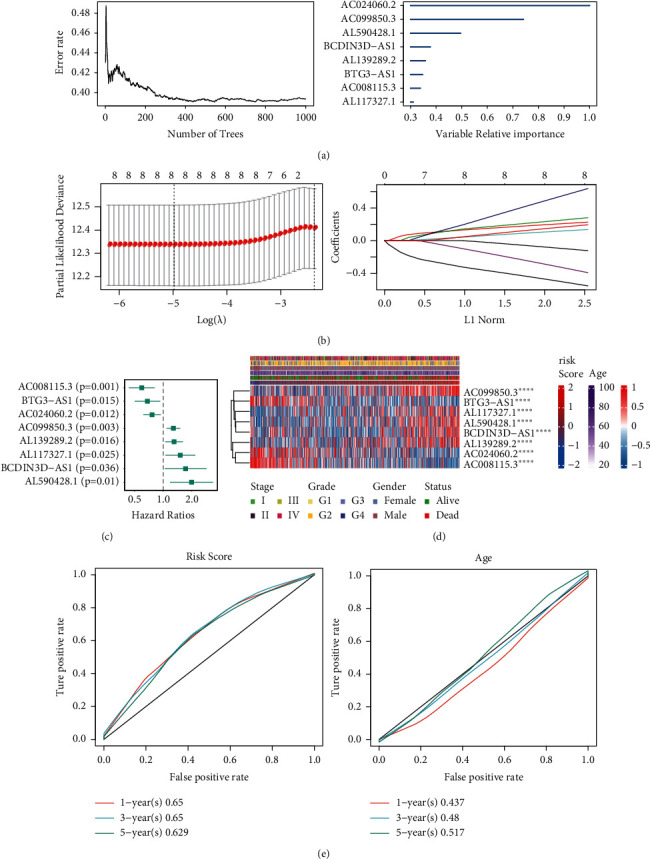
Screening of lncRNAs closely related to m6A. (a) The error rate of random forest classification for the top 2500 most important LncRNAs. (b) 3 low-risk genes and 5 high-risk genes were analyzed by Lasso regression. (c) Univariate regression was used to analyze the risk significance P values of 8 genes. (d) The correlation between 8 lncRNAs and the prognosis of HNSCC. (e) The ROC curve of risk score and age. ^*∗∗∗∗*^indicates that the gene is statistically significant in the survival model.

Studies have shown that m6A plays an important role in HNSCC [25]. To study m6A-related lncRNAs, we initially obtained 2444 m6A-related lncRNAs. Then, 442 lncRNAs were screened using single-factor analysis. Survival random forest analysis revealed eight lncRNAs that were closely linked to prognosis: AC008115.3, BTG3-AS1, AC024060.2, AC099850.3, AL117327.1, BCDIN3D-AS1, and AL590428.1 (Figure 1(a)). LASSO regression analysis was used to obtain a risk model containing eight genes, and the best log(*λ*) was −5. There were five high-risk and three low-risk genes (Figure 1(b)). Single-factor results of the eight genes in our survival model were significant (*P* < 0.05). These eight genes were significantly associated with HNSCC prognosis (Figure 1(c)), and they were linked to patient survival. Survival analysis of the eight genes was plotted (Figure S1). The survival model (TNM stage, grade, sex, age, status, and risk score) was related to the eight genes (Figure 1(d)). The ROC curve showed that the risk score had a strong predictive ability, with an AUC of 0.65, 0.65, and 0.629 in 1, 3, and 5 years compared with age factors. The risk model may serve as an important indicator for evaluating the prognosis of HNSCC (Figure 1(e)). Ultimately, we obtained eight lncRNAs closely related to m6A and HNSCC prognosis.

### 2.2. Prognostic Model of the m6A Gene and Its Clinical Characteristics

**Figure 2:**
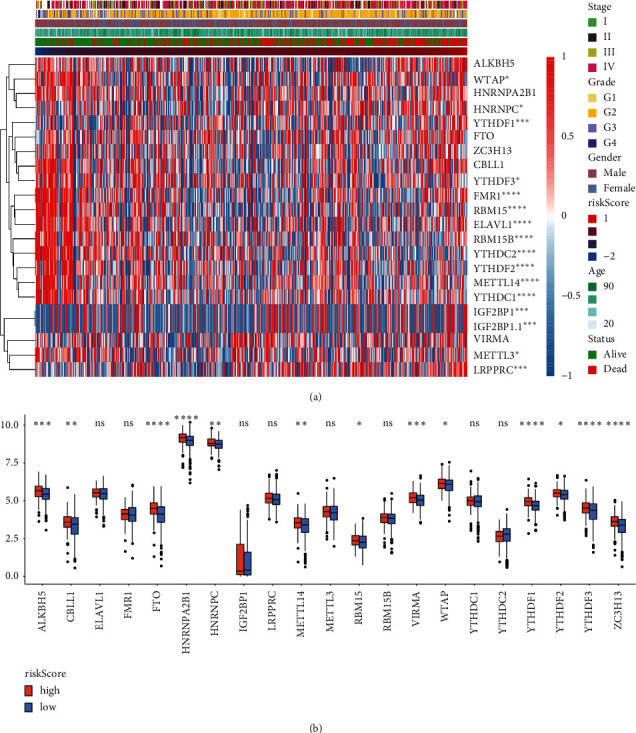
Correlation between the m6A gene and prognosis markers of HNSCC. (a) Heat map of variation in m6A gene expression across cancer stage, cancer grade, patient sex, patient age, status, and risk score. (b) The level of m6A (21 genes) expression in high- and low-risk-score groups. ^*∗*^, *P* < 0.05; ^*∗∗*^, *P* < 0.01; ^*∗∗∗*^, *P* < 0.001; ^*∗∗∗∗*^, *P* < 0.0001; ns, not significant.

We further explored the correlation between risk scores and m6A gene expression. We first selected 21 m6A RNA methylation regulators from previously published articles [26]. The results showed that the survival model (stage, grade, sex, age, status, and risk score) was correlated with the following genes: WTAP, HNRNPC, YTHDF1, FMR1, RBM15, ELAVL1, RBM15B, YTHDC2, YTHDF2, METL14, YTHDC1, IGF2BP1, IGF2BP1.1, MRTTL3, and LRPPRC (Figure 2(a)). We divided risk scores into high and low groups. Expression levels of ALKBH5, CBLL1, FMR1, HNRNPA2B1, HNRNPC, METTL14, MRTTL3, RBM15, ELAVL, VIRMA, WTAP, YTHDF1, YTHDF2, and YTHDF3 were significantly different in m6A modification (Figure 2(b)). In short, HNSCC prognosis is significantly correlated to the m6A gene.

### 2.3. Correlation between Risk Scores and Clinical Features

**Figure 3:**
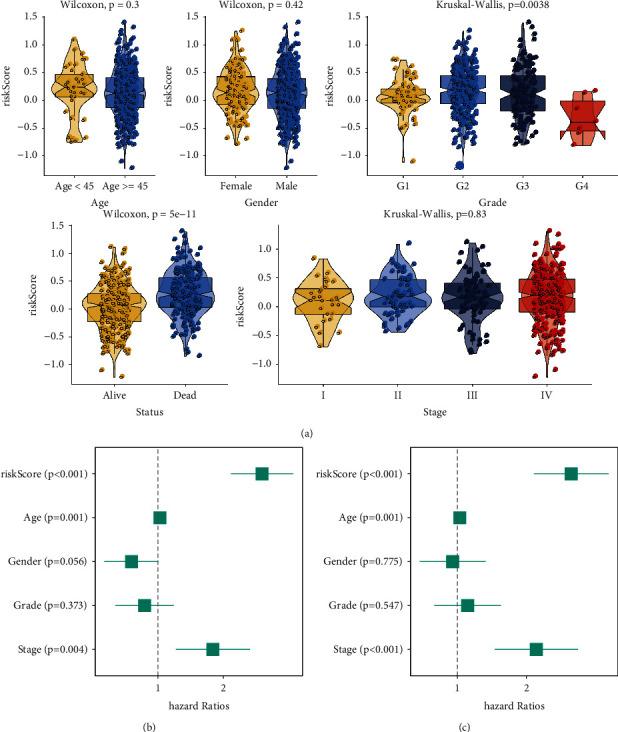
Clinical characteristics of risk scores. (a) Wilcoxon and Kruskal–Wallis tests were used to analyze risk score distribution in other clinical features (status, age, gender, grade, and stage). (b), (c) Univariate and multivariate analyses were performed on the prognostic factors (risk score, age, gender, grade, and stage).

The abovementioned experiment showed that m6A gene expression differed significantly across risk-score groups. Risk score data were then classified by age, sex, grade, status, and stage. Risk-score distributions were significantly different in grade and status (*P* < 0.05), but not in age, sex, and stage (Figure 3(a)). Univariate (Figure 3(b)) and multivariate analyses (Figure 3(c)) revealed that the risk score was significantly correlated with age and stage (*P* < 0.05). Specifically, stage, grade, and risk score all increased with increasing age.

### 2.4. Immunoinfiltration Analysis of the Prognosis Model

**Figure 4:**
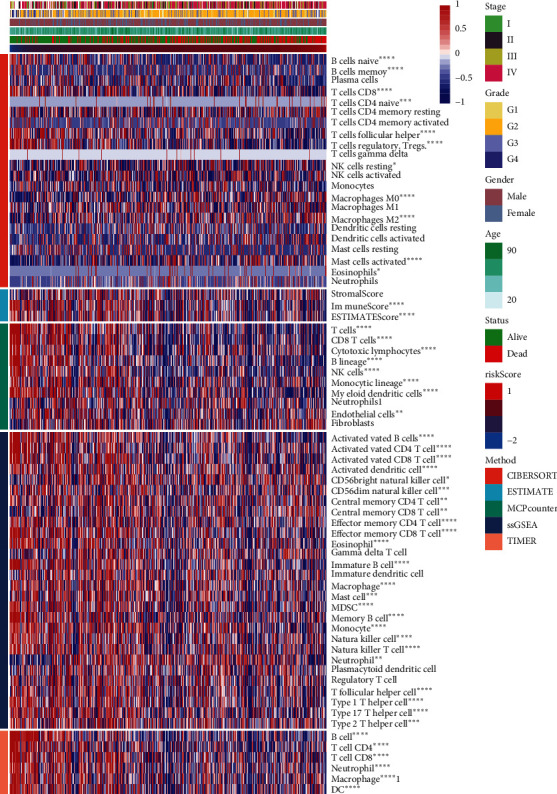
Link between prognostic models and immune cells. Correlation of the HNSCC prognosis model and 69-immune cell lineage genes. ^*∗*^, *P* < 0.05; ^*∗∗*^, *P* < 0.01; ^*∗∗∗*^, *P* < 0.001; ^*∗∗∗∗*^, *P* < 0.0001.

The abovementioned results analyzed clinical characteristics and then identified a correlation between prognosis and immune infiltration. As risk score increased, the proportion of the following immune cells and factors decreased: naïve B cells, memory B cells, follicular helper T cells, NK cells, activated B cells, effector memory CD4 T cells, effector memory CD8 T cells, memory B cells, CD4 T cells, and CD8 T cells. Likewise, stage, grade, disease severity, and immune system dysfunction increased with greater risk scores in HNSCC patients (Figure 4). B and T cells were abnormal. In conclusion, immune infiltration is associated with risk scores in the HNSCC prognosis model.

### 2.5. Immune Checkpoint Analysis of Different Risk-Score Groups

**Figure 5:**
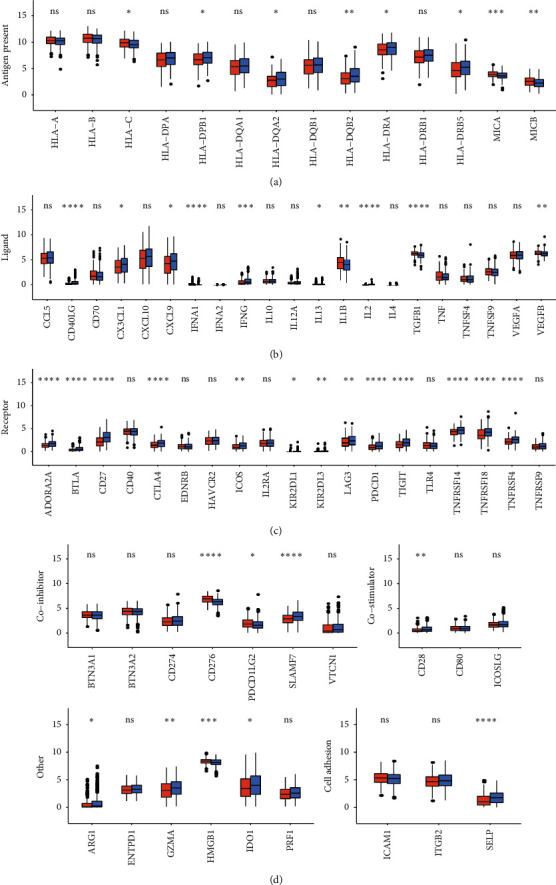
Immune checkpoint. (a) The level of 14 genes at different levels of antigen present. (b) Ligand gene expression under different risk scores. (c) Gene expression at different receptor levels in high- and low-risk-score groups. (d) Gene expression at different levels of coinhibitor, costimulator, cell adhesion, and “others.” ^*∗*^, *P* < 0.05; ^*∗∗*^, *P* < 0.01; ^*∗∗∗*^, *P* < 0.001; ^*∗∗∗∗*^, *P* < 0.0001; ns, not significant.

The abovementioned experiment showed the correlation between the prognostic model and immune cells. Immunotherapy, mediated by the immune checkpoint inhibitor (ICI), represents a turning point in the antitumor treatment of various cancer types in recent years [27]. We have analyzed the correlation between high and low risk score and immune checkpoints. Classification of immune inspection: antigen present, ligand, receptor, coinhibitor, costimulator, others, and cell adhesion. At the antigen present level (Figure 5(a)), the expression of HLA-DPB1, HLA-DQA2, HLA-DQB2, HLA-DRA, HLA-DRB5, MICA, and MICB increased in the high-risk-score group. At the ligand level (Figure 5(b)), the expression of CD40LG, CX3CL1, CXCL9, and IFNG increased in the high-risk-score group. The expression of TGFB1 and VEGFB decreased. At the receptor level (Figure 5(c)), the expression of ADORA2A, BTLA, CD27, CTLA4, ICOS, LAG3, TNFRSF14, TNFRSF18, TNFRSF4, and TNFRSF9 increased in the high-risk-score group. At the level of coinhibitor, costimulator, cell adhesion, and others (Figure 5(d)), the expression of CD276, PDCD1LG2, and HMGB1 decreased in the high-risk-score group. The expression of CD28, ARG1, GZMA, IDO1, and SELP increased. A scatter plot was used to analyze the correlation between risk score and several classical immune checkpoint molecules. The results showed that HLA-C (*r* = 0.16) and CD276 (*r* = 0.42) were positively correlated with risk score. CD40LG (*r* = 0.27), CD27 (*r* = 0.34), CD28 (*r* = 0.19), and SELP (*r* = 0.19) were negatively correlated with risk score (Figure S2). The abnormal expression of the abovementioned classic immune checkpoint genes has obvious correlation with high and low risk score.

### 2.6. Functional Analysis of Prognostic Models

**Figure 6:**
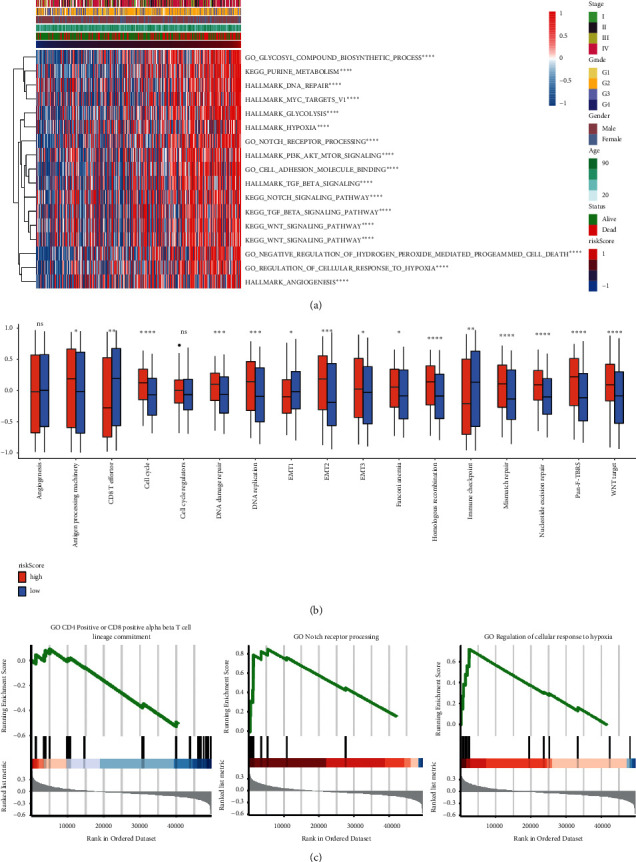
HNSCC prognosis is likely related to various signaling pathways. (a) Heat maps of signal pathways in clinical models. (b) Classical pathway expression differed across risk-score groups. (c) Enrichment plot for the gene set of dendrite. *Y*-axis: the value of the ranking metric; *X*-axis: the rank for all genes. Bottom: plot of the ranked list of all genes. ^*∗*^, *P* < 0.05; ^*∗∗*^, *P* < 0.01; ^*∗∗∗*^, *P* < 0.001; ^*∗∗∗∗*^, *P* < 0.0001; ns, not significant.

Our functional analysis of risk scores from the prognostic model revealed that the main pathways affecting prognosis were the notch, TGF-*β*, Wnt, and PI3K signaling pathways (Figure 6(a)). These pathways are primarily related to proliferation, apoptosis, cycle, and inflammatory responses of HNSCC cells. As risk score increased, survival decreased, while the expressions of CD8 T cells and immune checkpoints both rose (Figure 6(b)). Gene Set Enrichment Analysis (GSEA) revealed that when NES was positive, notch expression and regulation of the cellular response to hypoxia pathways increased significantly in the high group. However, when NES was negative, CD4 and CD8 expression decreased significantly in the low group (Figure 6(c)). In conclusion, HNSCC prognosis may be related to abnormal signaling pathways.

## 3. Materials and Methods

### 3.1. HNSCC Dataset and Preprocessing

RNA-sequencing data were downloaded from the TCGA data portal. The TCGA dataset was downloaded from UCSC Xena (https://xenabrowser.net/) (Supplementary Table S1). The number of segments per million value of each segment was converted to the transcript/points per million. We obtained 21 m6A genes, and Pearson correlations were used to screen related lncRNAs. Correlations were considered significant with a coefficient |R2| >0.3 and *P* < 0.05. Collected clinical pathological data included sex, age, stage, grade, survival status, and survival duration.

### 3.2. Establishment of m6A-Related lncRNA Risk Scores

Univariate Cox regression was used to screen m6A-related lncRNAs, and random survival forests were used for further screening. We used the LASSO method to select the highest lambda value (“min” lambda) of the selected genes after 1000 cross validations. We obtained a set of prognostic genes and their LASSO regression coefficients. The risk score was obtained from LASSO-screened genes, and its value was the sum of LASSO regression coefficients. The risk-score equation was as follows: risk score = −0.1069 *∗* AC024060.2 + 0.2167 *∗* AC099850.3 + 0.2651 *∗* AL590428.1 + 0.5882 *∗* BCDIN3D-AS1 + 0.1269 *∗* AL139289.2 + −0.3562 *∗* BTG3-AS1 + −0.5245 *∗* AC008115.3 + 0.1792 *∗* AL117327.1. According to the predictive model, the patients were divided into high-risk and low-risk groups using the median cutoff of risk score. The Cox proportional hazard regression model includes stage, grade, status, age, and TNM stage. The hazard ratio (HR) from Cox regression analysis was used to distinguish the prognostic factors positively or negatively. A gene with HR >1 was considered a risk gene, and a gene with HR <1 was considered a protective gene. Subsequently, the Kaplan–Meier survival method was used to evaluate the availability of the prognostic model, and the sensitivity and specificity of the receiver operating characteristic (ROC) curve were used to evaluate the prognostic accuracy of the signature building.

### 3.3. Analysis of the m6A Gene and Clinical Characteristics

We analyzed the correlation between prognostic score and M6a gene expression. Next, we examined variation in risk scores across different clinical features. Univariate and multivariate analyses were performed for risk scores and clinical characteristics.

### 3.4. Estimation of Immune Infiltration

The CiberSort, Estimate, McCounter, Single Sample Gene Set Enrichment Analysis (SSGSEA), and TIME algorithms were compared to evaluate the relationship between risk score and cell composition or cellular immune response. Heat maps were used to reveal differences in immune responses under various algorithms.

### 3.5. Pathway Analysis

All gene sets were downloaded from the Sigdb database. The GSVA software package was used to analyze immune checkpoint clusters and scores. This includes the GO BP (biological process), KEGG, and hallmark gene sets. The clusterProfiler package from R was utilized for GSEA analysis.

### 3.6. Statistical Analyses

All statistical analyses were performed in R (version 3.6.1, https://www.r-project.org/). The Wilcoxon and Kruskal–Wallis tests were used to compare nonnormally distributed (nonparametric) variables. Pearson and distance correlations were used to calculate correlation coefficients. Data were mainly visualized using the R package ggplot2. The Kaplan–Meier method was used to generate and visualize subgroup survival curves of. All tests were two sided. Significance was set at *P* < 0.05.

## 4. Discussion

This bioinformatics study found that lncRNAs are related to HNSCC prognosis. Risk score and m6A gene expression were correlated in the prognostic model. Disease grade and status differed significantly across risk-score groups. The proportion of immune cells and factors decreased with increasing risk score. Additionally, high and low risk scores are related to the expression of immune checkpoints. We suggest that HNSCC prognosis may be related to proliferation, apoptosis, cycle, and inflammatory response signaling pathways.

The lncRNA subclass eRNA is derived from the enhancer region of a gene; they are cis-acting sequences that affect transcription [28]. Our standards include functionally unannotated AC008115.3, BTG3-AS1, AC024060.2, AC099850.3, AL117327.1 BCDIN3D-AS1, and AL590428.1 identified as important eRNA candidates in HNSCC. Here, we determined prognostic eRNA and its target genes in HNSCC. We found that m6A and long noncoding lncRNAs were significantly associated with HNSCC patient survival. In HNSCC, m6A is related to the abnormal expression of other immune checkpoints (antigen presence, ligand, receptors, co-inhibitors, co-stimulators, and cell adhesion). The extent of discordance was an unanticipated result given there is usually significant collinearity between various adverse prognostic factors. However, this assessment fails to account for within-group heterogeneity and highlights the importance of also using objective measures of model performance when developing and validating risk score. Although we found risk score to be the better predictor of disease-specific survival than the age, the performance was modest at best with an AUC value of 0.65.

The immune microenvironment of HNSCC is characterized by immune cell populations, immune checkpoints, and changes in tumor or microenvironmental factors that are conducive to immune suppression. As a result, the tumor evades and escapes host immune surveillance [29]. Inflammation response is an essential component of the tumor microenvironment [30]. Increasing evidence has shown that immune cell dysfunction in HNSC-TME promotes immune suppression, thereby enhancing tumor survival and progression [31, 32]. Our analysis showed that the density of CD4+ T cells, CD8+ T cells, plasma cells, and M1 macrophages, along with higher immune scores, were associated with patient prognosis, consistent with previous studies [33]. The preexisting immune response has antitumor effects and positively affects response to immunotherapy. Some groundbreaking clinical and genomic studies have reported that HNSCC is a tumor with a high degree of immune cell infiltration [34–36]. However, less than 20% of patients with HNSC responded to immunotherapy, fewer than patients with other tumor types that have lower immunoinvasion rates [37]. This pattern suggests that even immunophenotypes in tumors cannot fully predict response to immunotherapy. Molecular analysis of HNSC identified a series of cytokines and chemokines that determine the host's ability to form an antitumor immune response. During tumorigenesis, these molecular changes may interfere with intercellular communication between infiltrating immune cells, thus destroying the balance between immune tolerance and activity [38]. The high-risk-score group tended to correlate with more immune infiltrating cells, such as macrophages and fibroblasts. The high-risk-score group also expressed more immune checkpoint molecules including PDCD1 and chemokines CCL-5, CXCL10, and CXCL9 [39]. Our results showed that higher risk scores were associated with lower numbers of immune cells, including naïve B cells, B-cell membranes, T cells, follicular helper T cells, NK cells, memory B cells, CD4 T cells, and CD8 T cells.

In conclusion, HNSCC exhibits significantly abnormal lncRNAs that negatively affect survival. The m6A gene differed significantly between high and low risk scores in the clinical model, suggesting that it can be used as a prognostic marker for HNSCC. Patients with higher risk scores have inactivated immune cells and abnormal expression of immune checkpoints. High-risk lncRNAs may interfere with m6A expression in HNSCC, altering the immune system and endangering patients.

## Data Availability

The data supporting the findings of this study are available from the corresponding author upon request.

## Supplementary Materials

Supplementary Materials Figure S1: the survival analysis of AC008115.3, BTG3-AS1, AC024060.2, AC099850.3, AL139289.2, AL117327.1, BCDIN3D-AS1, and AL590428.1. Figure S2: the correlation between risk score and 6 classical immune checkpoints (HLA-C, CD276, CD40LG, CD27, CD28, and SELP). Table S1: the TCGA dataset is downloaded from UCSC Xena.

## References

[1] Bray F., Ferlay J., Soerjomataram I., Siegel R. L., Torre L. A., Jemal A. (2018). Global cancer statistics 2018: GLOBOCAN estimates of incidence and mortality worldwide for 36 cancers in 185 countries. *CA: A Cancer Journal for Clinicians*.

[2] Yokota T., Homma A., Kiyota N. (2020). Immunotherapy for squamous cell carcinoma of the head and neck. *Japanese Journal of Clinical Oncology*.

[3] Chang J.-H., Wu C.-C., Yuan K. S.-P., Wu A. T. H., Wu S.-Y. (2017). Locoregionally recurrent head and neck squamous cell carcinoma: incidence, survival, prognostic factors, and treatment outcomes. *Oncotarget*.

[4] Bossi P., Alfieri S., Strojan P. (2019). Prognostic and predictive factors in recurrent and/or metastatic head and neck squamous cell carcinoma: a review of the literature. *Critical Reviews in Oncology*.

[5] Ho A. S., Kraus D. H., Ganly I., Lee N. Y., Shah J. P., Morris L. G. T. (2014). Decision making in the management of recurrent head and neck cancer. *Head & Neck*.

[6] Zandberg D. P., Strome S. E. (2014). The role of the PD-L1:PD-1 pathway in squamous cell carcinoma of the head and neck. *Oral Oncology*.

[7] Iyer M. K., Niknafs Y. S., Malik R. (2015). The landscape of long noncoding RNAs in the human transcriptome. *Nature Genetics*.

[8] Arun G., Diermeier S. D., Spector D. L. (2018). Therapeutic targeting of long non-coding RNAs in cancer. *Trends in Molecular Medicine*.

[9] Shih J. W., Chiang W. F., Wu A. T. H. (2017). Long noncoding RNA LncHIFCAR/MIR31HG is a HIF-1*α* co-activator driving oral cancer progression. *Nature Communications*.

[10] Bartonicek N., Maag J. L., Dinger M. E. (2016). Long noncoding RNAs in cancer: mechanisms of action and technological advancements. *Molecular Cancer*.

[11] Arunkumar G., Murugan A. K., Prasanna Srinivasa Rao H., Subbiah S., Rajaraman R., Munirajan A. K. (2017). Long non-coding RNA CCAT1 is overexpressed in oral squamous cell carcinomas and predicts poor prognosis. *Biomedical Reports*.

[12] Dorji T., Monti V., Fellegara G. (2015). Gain of hTERC: a genetic marker of malignancy in oral potentially malignant lesions. *Human Pathology*.

[13] Natoli G., Andrau J.-C. (2012). Noncoding transcription at enhancers: general principles and functional models. *Annual Review of Genetics*.

[14] Fang Y., Fullwood M. J. (2016). Roles, functions, and mechanisms of long non-coding RNAs in cancer. *Genomics, Proteomics & Bioinformatics*.

[15] Alarcón C. R., Lee H., Goodarzi H., Halberg N., Tavazoie S. F. (2015). N6-methyladenosine marks primary microRNAs for processing. *Nature*.

[16] Zhao B. S., Roundtree I. A., He C. (2016). Post-transcriptional gene regulation by mRNA modifications. *Nature Reviews Molecular Cell Biology*.

[17] Lan Q., Liu P. Y., Haase J., Bell J. L., Hüttelmaier S., Liu T. (2019). The critical role of RNA m6A methylation in cancer. *Cancer Research*.

[18] Seiwert T. Y., Burtness B., Mehra R. (2016). Safety and clinical activity of pembrolizumab for treatment of recurrent or metastatic squamous cell carcinoma of the head and neck (KEYNOTE-012): an open-label, multicentre, phase 1b trial. *The Lancet Oncology*.

[19] Prat A., Navarro A., Paré L. (2017). Immune-related gene expression profiling after PD-1 blockade in non-small cell lung carcinoma, head and neck squamous cell carcinoma, and melanoma. *Cancer Research*.

[20] Zeng D., Zhou R., Yu Y. (2018). Gene expression profiles for a prognostic immunoscore in gastric cancer. *British Journal of Surgery*.

[21] Jiang Y., Zhang Q., Hu Y. (2018). ImmunoScore signature. *Annals of Surgery*.

[22] Noy R., Pollard J. W. (2014). Tumor-associated macrophages: from mechanisms to therapy. *Immunity*.

[23] Vassilakopoulou M., Avgeris M., Velcheti V. (2016). Evaluation of PD-L1 expression and associated tumor-infiltrating lymphocytes in laryngeal squamous cell carcinoma. *Clinical Cancer Research*.

[24] Mei Z., Huang J., Qiao B., Lam A. K. (2020). Immune checkpoint pathways in immunotherapy for head and neck squamous cell carcinoma. *International Journal of Oral Science*.

[25] Zhao X., Cui L. (2019). Development and validation of a m6A RNA methylation regulators-based signature for predicting the prognosis of head and neck squamous cell carcinoma. *American journal of cancer research*.

[26] Jin Y., Wang Z. (2021). Analysis of m6A-related signatures in the tumor immune microenvironment and identification of clinical prognostic regulators in adrenocortical carcinoma. *Frontiers in Immunology*.

[27] Zhang H., Dai Z. (2021). Regulatory mechanisms of immune checkpoints PD-L1 and CTLA-4 in cancer. *Journal of Experimental & Clinical Cancer Research*.

[28] Gu X., Wang L., Boldrup L. (2019). AP001056.1, a prognosis-related enhancer RNA in squamous cell carcinoma of the head and neck. *Cancers*.

[29] Solomon B., Young R. J., Rischin D. (2018). Head and neck squamous cell carcinoma: genomics and emerging biomarkers for immunomodulatory cancer treatments. *Seminars in Cancer Biology*.

[30] Zhang H., He J. (2021). PDIA5 is correlated with immune infiltration and predicts poor prognosis in gliomas. *Frontiers in Immunology*.

[31] Rooney M. S., Shukla S. A., Wu C. J., Getz G., Hacohen N. (2015). Molecular and genetic properties of tumors associated with local immune cytolytic activity. *Cell*.

[32] Davis R. J., Van Waes C., Allen C. T. (2016). Overcoming barriers to effective immunotherapy: MDSCs, TAMs, and Tregs as mediators of the immunosuppressive microenvironment in head and neck cancer. *Oral Oncology*.

[33] He Y., Jiang Z., Chen C., Wang X. (2018). Classification of triple-negative breast cancers based on immunogenomic profiling. *Journal of Experimental & Clinical Cancer Research: Climate Research*.

[34] Şenbabaoğlu Y., Gejman R. S., Winer A. G. (2016). Tumor immune microenvironment characterization in clear cell renal cell carcinoma identifies prognostic and immunotherapeutically relevant messenger RNA signatures. *Genome Biology*.

[35] Yoshihara K., Shahmoradgoli M., Martínez E. (2013). Inferring tumour purity and stromal and immune cell admixture from expression data. *Nature Communications*.

[36] Mandal R., Şenbabaoğlu Y., Desrichard A. (2016). The head and neck cancer immune landscape and its immunotherapeutic implications. *JCI Insight*.

[37] Yarchoan M., Hopkins A., Jaffee E. M. (2017). Tumor mutational burden and response rate to PD-1 inhibition. *New England Journal of Medicine*.

[38] Chen D. S., Mellman I. (2017). Elements of cancer immunity and the cancer-immune set point. *Nature*.

[39] Zhang N., Dai Z. (2021). The predictive value of monocytes in immune microenvironment and prognosis of glioma patients based on machine learning. *Frontiers in Immunology*.

