# Supplementary material for: Gene Model Related to m6A Predicts the Prognostic Effect of Immune Infiltration on Head and Neck Squamous Cell Carcinoma
Source: J Oncol. 2021 Aug 20;2021:1814266. doi: 10.1155/2021/1814266 (PMC8397575; doi:10.1155/2021/1814266)
Supplement: Supplementary Materials — Figure S1: the survival analysis of AC008115.3, BTG3-AS1, AC024060.2, AC099850.3, AL139289.2, AL117327.1, BCDIN3D-AS1, and AL590428.1. Figure S2: the correlation between risk score and 6 classical immune checkpoints (HLA-C, CD276, CD40LG, CD27, CD28, and SELP). Table S1: the TCGA dataset is downloaded from UCSC Xena. [file 1814266.f1.zip › 1814266.f1/table S1 (1).docx]

**Table S1** **The TCGA dataset is downloaded from UCSC Xena (https://xenabrowser.net/) (Supplementary)**

| **gene** | **HR** | **lower.95** | **upper.95** | **pvalue** |
| --- | --- | --- | --- | --- |
| AC005225.4 | 0.331223 | 0.117581 | 0.933048 | 0.036518 |
| AC005562.1 | 1.264058 | 1.003618 | 1.592082 | 0.04652 |
| AC005696.1 | 0.744198 | 0.587179 | 0.943206 | 0.014543 |
| AC005726.4 | 0.786516 | 0.627348 | 0.986066 | 0.037379 |
| AC007823.1 | 2.26606 | 1.117589 | 4.594736 | 0.023316 |
| AC008581.1 | 0.859125 | 0.738907 | 0.998902 | 0.048353 |
| AC009303.4 | 1.470651 | 1.034679 | 2.090324 | 0.031556 |
| AC010834.3 | 0.462571 | 0.252951 | 0.845902 | 0.012302 |
| AC012467.2 | 0.66816 | 0.464172 | 0.961793 | 0.03004 |
| AC013652.1 | 1.318843 | 1.089736 | 1.596118 | 0.004474 |
| AC015909.5 | 0.249854 | 0.072566 | 0.860274 | 0.02791 |
| AC037459.2 | 0.666234 | 0.481033 | 0.922739 | 0.014532 |
| AC060780.1 | 0.654602 | 0.435327 | 0.984327 | 0.041764 |
| AC087500.1 | 0.490683 | 0.284689 | 0.84573 | 0.010371 |
| AC087501.4 | 0.500101 | 0.257335 | 0.971888 | 0.040946 |
| AC092295.2 | 0.657491 | 0.466395 | 0.926886 | 0.016697 |
| AC093752.3 | 0.639503 | 0.462071 | 0.885067 | 0.007011 |
| AC099850.3 | 1.282717 | 1.086371 | 1.51455 | 0.003311 |
| AC104083.1 | 0.832949 | 0.712887 | 0.973232 | 0.021356 |
| AC106820.5 | 0.349432 | 0.181451 | 0.672921 | 0.001662 |
| AC108010.1 | 0.816817 | 0.680855 | 0.979931 | 0.029391 |
| AC114271.1 | 0.610425 | 0.414939 | 0.898008 | 0.012205 |
| AC127496.6 | 0.772388 | 0.631393 | 0.944869 | 0.012025 |
| AL135925.1 | 1.526062 | 1.085392 | 2.145646 | 0.015045 |
| AL662795.2 | 0.693453 | 0.532636 | 0.902824 | 0.006541 |
| AP003392.3 | 0.80695 | 0.662028 | 0.983597 | 0.033694 |
| BCDIN3D-AS1 | 1.723191 | 1.036265 | 2.86547 | 0.035971 |
| FOXD2-AS1 | 1.342978 | 1.05191 | 1.714586 | 0.017981 |
| GAS5-AS1 | 0.612152 | 0.412916 | 0.907522 | 0.014566 |
| GLIDR | 1.386706 | 1.06277 | 1.809377 | 0.01602 |
| JPX | 1.382513 | 1.089534 | 1.754274 | 0.007683 |
| LINC00324 | 0.734978 | 0.566669 | 0.953279 | 0.02031 |
| LINC01719 | 1.524628 | 1.062792 | 2.187154 | 0.021979 |
| LIPE-AS1 | 0.855358 | 0.748347 | 0.977672 | 0.021958 |
| MAP4K3-DT | 1.358629 | 1.047878 | 1.761534 | 0.020728 |
| MSC-AS1 | 1.155264 | 1.02737 | 1.29908 | 0.015907 |
| NKILA | 1.225502 | 1.035453 | 1.450434 | 0.018022 |
| TTN-AS1 | 1.093644 | 1.012361 | 1.181453 | 0.023102 |
| AC005306.1 | 0.722913 | 0.56087 | 0.931772 | 0.012221 |
| AC005410.2 | 0.284296 | 0.091454 | 0.883771 | 0.029744 |
| AC007114.2 | 0.010083 | 0.000209 | 0.486306 | 0.020098 |
| AC007191.1 | 0.596239 | 0.384275 | 0.925121 | 0.021042 |
| AC007283.1 | 0.829009 | 0.703019 | 0.977577 | 0.025774 |
| AC007787.2 | 0.28657 | 0.084427 | 0.972709 | 0.045034 |
| AC007878.1 | 0.135762 | 0.038097 | 0.483794 | 0.002071 |
| AC008074.2 | 0.653762 | 0.460855 | 0.927418 | 0.017203 |
| AC008764.9 | 0.075859 | 0.012912 | 0.445671 | 0.00431 |
| AC008993.1 | 0.588201 | 0.384475 | 0.899879 | 0.014435 |
| AC009034.1 | 0.537423 | 0.365786 | 0.789597 | 0.001559 |
| AC009090.4 | 0.1495 | 0.03491 | 0.640227 | 0.010442 |
| AC009095.1 | 0.050218 | 0.008497 | 0.296783 | 0.000967 |
| AC009630.3 | 0.818273 | 0.681477 | 0.982529 | 0.03165 |
| AC011476.3 | 0.739331 | 0.589335 | 0.927503 | 0.009042 |
| AC011815.3 | 0.551456 | 0.304794 | 0.997735 | 0.04913 |
| AC012313.5 | 0.423894 | 0.21986 | 0.817275 | 0.010396 |
| AC012513.3 | 0.535003 | 0.370856 | 0.771804 | 0.000822 |
| AC012531.1 | 0.370394 | 0.139298 | 0.984877 | 0.046535 |
| AC012615.6 | 0.531111 | 0.31012 | 0.909582 | 0.021155 |
| AC012676.5 | 0.505113 | 0.31171 | 0.818514 | 0.005552 |
| AC015849.3 | 0.440092 | 0.247976 | 0.781046 | 0.005043 |
| AC018665.1 | 0.69037 | 0.521388 | 0.914118 | 0.009685 |
| AC022150.4 | 0.701601 | 0.519051 | 0.948354 | 0.021176 |
| AC022167.2 | 0.766781 | 0.603064 | 0.974943 | 0.030232 |
| AC024075.1 | 0.612781 | 0.410485 | 0.914774 | 0.016588 |
| AC025917.1 | 0.464748 | 0.251617 | 0.858411 | 0.014379 |
| AC027097.1 | 0.713439 | 0.512913 | 0.992363 | 0.04491 |
| AC053513.2 | 0.194622 | 0.051701 | 0.732633 | 0.015522 |
| AC055811.4 | 0.689019 | 0.47881 | 0.991513 | 0.044871 |
| AC069185.1 | 0.565941 | 0.334149 | 0.958522 | 0.034214 |
| AC084018.2 | 0.418997 | 0.223014 | 0.787211 | 0.00686 |
| AC090181.3 | 0.149184 | 0.025873 | 0.860191 | 0.0333 |
| AC090587.2 | 0.636891 | 0.446876 | 0.9077 | 0.012572 |
| AC091132.3 | 0.428989 | 0.21919 | 0.8396 | 0.013502 |
| AC092139.1 | 0.756327 | 0.573135 | 0.998074 | 0.048429 |
| AC093278.2 | 0.634832 | 0.474845 | 0.848723 | 0.002162 |
| AC093525.7 | 0.691877 | 0.501307 | 0.954892 | 0.025042 |
| AC093525.9 | 0.291776 | 0.08925 | 0.953876 | 0.04154 |
| AC093627.4 | 0.724304 | 0.576846 | 0.909457 | 0.005484 |
| AC096536.2 | 1.434149 | 1.073783 | 1.915455 | 0.014602 |
| AC098484.1 | 0.623088 | 0.401946 | 0.965896 | 0.034421 |
| AC107068.1 | 0.529392 | 0.306467 | 0.914474 | 0.022576 |
| AC108704.2 | 0.353785 | 0.127705 | 0.9801 | 0.045649 |
| AC116366.1 | 0.479579 | 0.273016 | 0.84243 | 0.010574 |
| AC117503.4 | 0.517032 | 0.301447 | 0.886798 | 0.016557 |
| AC118344.1 | 0.125066 | 0.030648 | 0.510355 | 0.003762 |
| AC118344.2 | 0.23684 | 0.08906 | 0.629831 | 0.003897 |
| AC120114.3 | 0.489802 | 0.297082 | 0.807542 | 0.005144 |
| AC122129.1 | 0.413268 | 0.209413 | 0.815568 | 0.010842 |
| AC131649.2 | 0.497418 | 0.271158 | 0.912472 | 0.024079 |
| AC131971.1 | 0.199777 | 0.057878 | 0.689567 | 0.010834 |
| AC139530.1 | 0.792448 | 0.639689 | 0.981687 | 0.033243 |
| AC141586.5 | 0.027435 | 0.001301 | 0.578457 | 0.020784 |
| AC144548.1 | 0.574131 | 0.369535 | 0.892002 | 0.013574 |
| AC145098.1 | 0.626328 | 0.411218 | 0.953963 | 0.029294 |
| AC233300.1 | 0.362133 | 0.138758 | 0.945097 | 0.037955 |
| AC240565.1 | 0.670663 | 0.48934 | 0.919174 | 0.012991 |
| AC243919.2 | 0.641593 | 0.461312 | 0.892326 | 0.008368 |
| AC245041.1 | 1.11723 | 1.013495 | 1.231583 | 0.025776 |
| AC245060.6 | 0.220666 | 0.071919 | 0.677065 | 0.008248 |
| AF131215.5 | 0.700341 | 0.538928 | 0.910099 | 0.007706 |
| AL021707.6 | 0.84219 | 0.719134 | 0.986303 | 0.033081 |
| AL031282.2 | 0.572398 | 0.403249 | 0.8125 | 0.001798 |
| AL031320.2 | 0.06232 | 0.007233 | 0.536955 | 0.011541 |
| AL031846.2 | 0.217977 | 0.087366 | 0.543845 | 0.001092 |
| AL050331.2 | 0.58502 | 0.353112 | 0.969233 | 0.037408 |
| AL050343.2 | 0.600045 | 0.419397 | 0.858504 | 0.005193 |
| AL133355.1 | 0.577857 | 0.396035 | 0.843154 | 0.004441 |
| AL133406.2 | 0.409443 | 0.178221 | 0.94065 | 0.035367 |
| AL158212.3 | 0.714727 | 0.52593 | 0.971298 | 0.031869 |
| AL160006.1 | 0.730103 | 0.571438 | 0.932822 | 0.011862 |
| AL162595.1 | 0.827194 | 0.686966 | 0.996046 | 0.04531 |
| AL353593.1 | 0.464482 | 0.24729 | 0.872434 | 0.017113 |
| AL353763.1 | 0.233992 | 0.096572 | 0.566954 | 0.001297 |
| AL354733.3 | 0.749389 | 0.605215 | 0.927907 | 0.008138 |
| AL357033.3 | 0.674549 | 0.494649 | 0.919877 | 0.012859 |
| AL590428.1 | 1.992503 | 1.181303 | 3.360753 | 0.009748 |
| AL731571.1 | 0.30771 | 0.127431 | 0.743033 | 0.008785 |
| BMS1P4 | 0.446818 | 0.24358 | 0.819631 | 0.009254 |
| BTG3-AS1 | 0.681715 | 0.500243 | 0.929019 | 0.015258 |
| BX571846.1 | 0.633127 | 0.441121 | 0.908707 | 0.013167 |
| C2CD4D-AS1 | 0.817982 | 0.716897 | 0.933319 | 0.002833 |
| CCDC183-AS1 | 0.562598 | 0.356766 | 0.887182 | 0.013321 |
| CYP51A1-AS1 | 15.33601 | 1.788759 | 131.4839 | 0.01276 |
| DTX2P1-UPK3BP1-PMS2P11 | 0.821951 | 0.697128 | 0.969124 | 0.01964 |
| FAM215B | 0.549721 | 0.37837 | 0.798672 | 0.001692 |
| FBXL19-AS1 | 0.538707 | 0.327608 | 0.885832 | 0.014781 |
| LINC00957 | 0.759457 | 0.593523 | 0.971782 | 0.028704 |
| LINC01123 | 1.44654 | 1.201794 | 1.741129 | 9.50E-05 |
| LINC01197 | 0.581572 | 0.35375 | 0.956115 | 0.032607 |
| LINC01232 | 1.388052 | 1.137837 | 1.693289 | 0.001224 |
| LINC01772 | 0.363577 | 0.149145 | 0.886305 | 0.026052 |
| LINC01963 | 0.666836 | 0.523399 | 0.849581 | 0.001041 |
| LINC02541 | 1.284834 | 1.116217 | 1.478922 | 0.00048 |
| MIR193BHG | 1.207348 | 1.054335 | 1.382569 | 0.006426 |
| NPTN-IT1 | 0.536943 | 0.311831 | 0.924566 | 0.024907 |
| PLBD1-AS1 | 0.785838 | 0.628915 | 0.981917 | 0.033961 |
| STARD4-AS1 | 0.555934 | 0.357833 | 0.863706 | 0.009007 |
| Z94721.2 | 0.089791 | 0.012858 | 0.627024 | 0.01507 |
| AC002350.2 | 0.525518 | 0.341085 | 0.809677 | 0.003531 |
| AC004623.1 | 0.586168 | 0.344243 | 0.998112 | 0.049192 |
| AC004908.1 | 0.599134 | 0.411493 | 0.872339 | 0.007529 |
| AC007292.1 | 0.553085 | 0.366787 | 0.834007 | 0.004711 |
| AC008115.3 | 0.594982 | 0.434423 | 0.814884 | 0.001214 |
| AC008406.3 | 0.643108 | 0.456177 | 0.906639 | 0.011758 |
| AC008543.1 | 0.447281 | 0.214529 | 0.932556 | 0.031855 |
| AC008669.1 | 0.54436 | 0.325307 | 0.910918 | 0.020604 |
| AC008763.1 | 0.597139 | 0.425017 | 0.838965 | 0.002958 |
| AC008770.3 | 0.352641 | 0.152043 | 0.817896 | 0.01517 |
| AC009121.2 | 0.357091 | 0.153879 | 0.828661 | 0.016505 |
| AC009126.1 | 0.591672 | 0.355181 | 0.985626 | 0.043845 |
| AC009318.1 | 0.451535 | 0.24207 | 0.842252 | 0.01243 |
| AC010319.4 | 0.609915 | 0.381589 | 0.974861 | 0.038794 |
| AC011477.3 | 0.649559 | 0.424061 | 0.994966 | 0.04735 |
| AC011484.1 | 0.55935 | 0.389963 | 0.802313 | 0.001596 |
| AC011498.2 | 0.435978 | 0.19327 | 0.983475 | 0.045488 |
| AC011498.7 | 0.587405 | 0.430309 | 0.801854 | 0.000806 |
| AC012074.1 | 0.465017 | 0.2622 | 0.824717 | 0.008814 |
| AC012313.6 | 0.337391 | 0.157931 | 0.720776 | 0.005026 |
| AC012615.1 | 0.796919 | 0.65422 | 0.970743 | 0.024138 |
| AC016586.1 | 0.295012 | 0.121878 | 0.714093 | 0.006798 |
| AC020907.1 | 0.798761 | 0.676508 | 0.943106 | 0.008023 |
| AC022075.1 | 0.808264 | 0.681901 | 0.958043 | 0.014123 |
| AC022150.2 | 0.543907 | 0.334902 | 0.883347 | 0.013844 |
| AC024075.2 | 0.620504 | 0.480722 | 0.80093 | 0.000248 |
| AC025048.2 | 0.703793 | 0.496821 | 0.996989 | 0.048049 |
| AC026704.1 | 0.357687 | 0.183827 | 0.695977 | 0.002469 |
| AC046185.3 | 0.71385 | 0.589881 | 0.863873 | 0.000533 |
| AC068473.3 | 0.727346 | 0.545618 | 0.969602 | 0.029975 |
| AC073896.3 | 0.46986 | 0.228306 | 0.966985 | 0.040254 |
| AC079296.1 | 0.10552 | 0.019686 | 0.565589 | 0.008659 |
| AC084018.1 | 0.661498 | 0.475717 | 0.919832 | 0.01402 |
| AC084824.3 | 0.347446 | 0.137391 | 0.878653 | 0.025532 |
| AC092683.1 | 0.821614 | 0.72467 | 0.931526 | 0.002161 |
| AC092683.2 | 0.209398 | 0.085171 | 0.514819 | 0.000658 |
| AC096992.2 | 0.645542 | 0.472026 | 0.882842 | 0.006142 |
| AC099568.2 | 0.779875 | 0.626658 | 0.970553 | 0.025895 |
| AC104794.3 | 0.719253 | 0.585947 | 0.882887 | 0.001627 |
| AC115284.2 | 0.576352 | 0.353919 | 0.938581 | 0.026779 |
| AC124045.1 | 0.48988 | 0.280293 | 0.856184 | 0.012244 |
| AC132938.5 | 0.425269 | 0.189415 | 0.954803 | 0.038261 |
| AC146944.4 | 0.785261 | 0.65241 | 0.945165 | 0.010578 |
| AC232271.1 | 0.546084 | 0.336235 | 0.886902 | 0.014484 |
| AL021368.2 | 0.477435 | 0.305514 | 0.746102 | 0.001171 |
| AL021707.3 | 0.651218 | 0.449309 | 0.943859 | 0.023507 |
| AL117339.4 | 0.002258 | 1.63E-05 | 0.312182 | 0.015399 |
| AL121832.3 | 0.32175 | 0.113776 | 0.909883 | 0.032515 |
| AL354892.2 | 0.681086 | 0.471095 | 0.984681 | 0.041147 |
| AL683813.1 | 0.363271 | 0.14225 | 0.927702 | 0.034273 |
| AL928970.1 | 0.319921 | 0.131515 | 0.778237 | 0.011979 |
| KLF3-AS1 | 0.517506 | 0.333985 | 0.801868 | 0.003196 |
| LINC00342 | 0.761663 | 0.641631 | 0.90415 | 0.001861 |
| LINC01139 | 0.888043 | 0.793112 | 0.994336 | 0.039548 |
| LINC01213 | 1.336918 | 1.000795 | 1.785931 | 0.049375 |
| MIR600HG | 0.70758 | 0.544753 | 0.919077 | 0.009531 |
| MIR9-3HG | 0.794113 | 0.707896 | 0.890831 | 8.40E-05 |
| POC1B-AS1 | 0.21844 | 0.055683 | 0.856921 | 0.029155 |
| RAD51-AS1 | 0.763424 | 0.584898 | 0.99644 | 0.047011 |
| RAMP2-AS1 | 0.627275 | 0.444442 | 0.885321 | 0.007982 |
| RRN3P2 | 0.563221 | 0.328401 | 0.965947 | 0.036992 |
| SLX1A-SULT1A3 | 0.851067 | 0.725191 | 0.998791 | 0.048293 |
| TNRC6C-AS1 | 0.79772 | 0.681469 | 0.933801 | 0.004919 |
| WEE2-AS1 | 0.553483 | 0.404142 | 0.75801 | 0.000227 |
| Z92544.2 | 0.44781 | 0.286494 | 0.699959 | 0.000423 |
| AC002128.1 | 0.447599 | 0.218993 | 0.914843 | 0.027525 |
| AC003070.1 | 0.732957 | 0.563494 | 0.953383 | 0.020568 |
| AC004148.1 | 0.765972 | 0.5949 | 0.986237 | 0.038694 |
| AC004461.2 | 0.267236 | 0.086237 | 0.828133 | 0.02221 |
| AC004494.1 | 0.077011 | 0.013703 | 0.432806 | 0.003606 |
| AC004584.3 | 0.012373 | 0.000679 | 0.225361 | 0.003015 |
| AC005695.1 | 0.010815 | 0.000588 | 0.198775 | 0.002306 |
| AC006128.1 | 0.790281 | 0.633004 | 0.986634 | 0.037634 |
| AC006299.1 | 0.172859 | 0.055689 | 0.536552 | 0.002387 |
| AC007728.2 | 0.495893 | 0.247986 | 0.991629 | 0.047285 |
| AC008555.2 | 0.013116 | 0.000233 | 0.737056 | 0.034997 |
| AC010226.1 | 0.565423 | 0.387614 | 0.824798 | 0.003078 |
| AC011442.1 | 0.271989 | 0.106749 | 0.693009 | 0.006363 |
| AC011466.3 | 0.087922 | 0.01463 | 0.528391 | 0.007881 |
| AC011676.5 | 0.008642 | 0.000286 | 0.261446 | 0.006312 |
| AC012254.3 | 0.075569 | 0.010962 | 0.520944 | 0.008742 |
| AC012313.10 | 0.429863 | 0.204427 | 0.903902 | 0.025988 |
| AC013403.2 | 0.346683 | 0.185795 | 0.646891 | 0.000873 |
| AC015802.4 | 0.651476 | 0.435082 | 0.975496 | 0.037488 |
| AC015911.3 | 0.355545 | 0.208926 | 0.605058 | 0.000138 |
| AC019171.1 | 0.759247 | 0.629759 | 0.91536 | 0.00389 |
| AC020917.4 | 0.402976 | 0.214349 | 0.757596 | 0.004774 |
| AC023510.2 | 0.185616 | 0.06838 | 0.503854 | 0.000949 |
| AC024267.3 | 0.207073 | 0.073083 | 0.586722 | 0.003043 |
| AC025287.3 | 0.546812 | 0.332456 | 0.899377 | 0.017421 |
| AC026362.1 | 0.41897 | 0.234984 | 0.74701 | 0.003193 |
| AC027117.1 | 0.526487 | 0.296998 | 0.933303 | 0.028072 |
| AC027601.2 | 0.422127 | 0.184161 | 0.967585 | 0.041567 |
| AC073575.2 | 0.65602 | 0.433013 | 0.993878 | 0.046708 |
| AC079336.7 | 0.060903 | 0.004779 | 0.776156 | 0.031153 |
| AC079921.2 | 0.669819 | 0.463246 | 0.968508 | 0.033168 |
| AC087222.1 | 0.357685 | 0.188021 | 0.68045 | 0.001728 |
| AC087741.1 | 0.770122 | 0.607882 | 0.975661 | 0.030456 |
| AC090948.2 | 0.565233 | 0.329999 | 0.968148 | 0.037723 |
| AC091185.1 | 0.436302 | 0.193574 | 0.983393 | 0.045462 |
| AC092535.2 | 0.010225 | 0.000399 | 0.26203 | 0.005619 |
| AC104825.1 | 0.66347 | 0.495796 | 0.887848 | 0.005775 |
| AC106820.4 | 0.476146 | 0.262394 | 0.864023 | 0.014659 |
| AC109460.3 | 0.427282 | 0.243844 | 0.748716 | 0.002967 |
| AC116158.1 | 0.065688 | 0.005434 | 0.794009 | 0.032245 |
| AC124283.5 | 0.106319 | 0.014728 | 0.767487 | 0.026259 |
| AC127070.4 | 0.075204 | 0.011501 | 0.491736 | 0.006916 |
| AC133065.3 | 0.731204 | 0.593755 | 0.900472 | 0.003211 |
| AC135048.4 | 0.32189 | 0.146156 | 0.708922 | 0.004894 |
| AC136475.3 | 0.851932 | 0.747691 | 0.970706 | 0.016109 |
| AD000813.1 | 0.042753 | 0.003096 | 0.590421 | 0.018606 |
| ADORA2A-AS1 | 0.604457 | 0.377493 | 0.96788 | 0.036093 |
| AF129075.1 | 0.376929 | 0.150776 | 0.942299 | 0.036879 |
| AL022328.2 | 0.697359 | 0.496276 | 0.979917 | 0.037815 |
| AL031714.1 | 0.652096 | 0.47399 | 0.897128 | 0.008616 |
| AL035530.2 | 0.373242 | 0.153184 | 0.90943 | 0.03009 |
| AL035587.1 | 0.577542 | 0.350815 | 0.950799 | 0.030903 |
| AL049776.1 | 0.222911 | 0.058659 | 0.847085 | 0.027552 |
| AL109806.1 | 0.046435 | 0.002923 | 0.737772 | 0.029593 |
| AL109955.1 | 0.262755 | 0.102065 | 0.676436 | 0.005602 |
| AL121772.3 | 0.658245 | 0.455141 | 0.951982 | 0.026326 |
| AL121839.2 | 0.3058 | 0.14555 | 0.642483 | 0.00176 |
| AL121992.1 | 0.014754 | 0.000687 | 0.316816 | 0.007048 |
| AL139246.2 | 0.049733 | 0.002848 | 0.868409 | 0.039719 |
| AL157392.3 | 0.715557 | 0.592283 | 0.864489 | 0.000522 |
| AL355075.2 | 0.61175 | 0.382801 | 0.977632 | 0.039924 |
| AL355488.1 | 0.742756 | 0.568789 | 0.969931 | 0.028948 |
| AL356481.3 | 0.308987 | 0.134172 | 0.711573 | 0.00579 |
| AL445931.1 | 0.059757 | 0.01126 | 0.317144 | 0.000938 |
| AL512770.1 | 0.447549 | 0.228136 | 0.877984 | 0.019364 |
| AL513190.1 | 0.301391 | 0.167595 | 0.542002 | 6.20E-05 |
| AL662844.3 | 0.421932 | 0.191953 | 0.927451 | 0.031762 |
| AL662844.4 | 0.614886 | 0.391779 | 0.965046 | 0.034458 |
| AL732314.6 | 0.466955 | 0.261353 | 0.834299 | 0.010118 |
| ALMS1-IT1 | 1.9301 | 1.331067 | 2.798721 | 0.000524 |
| AP000692.1 | 0.522316 | 0.28668 | 0.951633 | 0.033843 |
| AP001056.1 | 0.44327 | 0.244855 | 0.802469 | 0.007217 |
| AP001178.3 | 0.05801 | 0.003664 | 0.918461 | 0.04335 |
| AP4B1-AS1 | 0.26517 | 0.094216 | 0.746318 | 0.011931 |
| BHLHE40-AS1 | 0.621716 | 0.435174 | 0.888222 | 0.009022 |
| C5orf66 | 0.742429 | 0.607579 | 0.907209 | 0.003589 |
| CARD8-AS1 | 0.705577 | 0.524712 | 0.948786 | 0.021006 |
| CTBP1-AS | 0.264152 | 0.110309 | 0.632551 | 0.002809 |
| EDRF1-AS1 | 0.346624 | 0.134055 | 0.896259 | 0.028821 |
| EGOT | 0.447565 | 0.238516 | 0.839838 | 0.012297 |
| EP300-AS1 | 0.490044 | 0.284631 | 0.843699 | 0.010079 |
| EPB41L4A-DT | 0.570312 | 0.385661 | 0.843372 | 0.004903 |
| GUSBP11 | 0.819668 | 0.686309 | 0.97894 | 0.028171 |
| HCG27 | 0.570515 | 0.374021 | 0.870239 | 0.009184 |
| HEXD-IT1 | 0.010204 | 0.0006 | 0.173528 | 0.001517 |
| LINC002481 | 0.233724 | 0.09472 | 0.576717 | 0.001609 |
| LINC00921 | 0.572545 | 0.347663 | 0.94289 | 0.028451 |
| LINC01237 | 0.459599 | 0.244467 | 0.864046 | 0.015793 |
| LINC01305 | 0.574279 | 0.422908 | 0.779831 | 0.000381 |
| LINC01355 | 0.582366 | 0.397398 | 0.853427 | 0.005557 |
| LINC01409 | 0.615836 | 0.449572 | 0.843591 | 0.002533 |
| MANEA-DT | 0.374947 | 0.145429 | 0.966693 | 0.04235 |
| MIAT | 0.789125 | 0.666142 | 0.934814 | 0.006148 |
| N4BP2L2-IT2 | 0.480544 | 0.234164 | 0.986155 | 0.04572 |
| NADK2-AS1 | 0.154805 | 0.035572 | 0.673694 | 0.012905 |
| NARF-IT1 | 0.335775 | 0.142331 | 0.792131 | 0.012699 |
| PSMD6-AS2 | 0.122773 | 0.033695 | 0.447339 | 0.001476 |
| PTOV1-AS2 | 0.79271 | 0.677368 | 0.927692 | 0.003786 |
| RBFADN | 0.487361 | 0.261097 | 0.909703 | 0.023998 |
| RFPL1S | 0.479187 | 0.279476 | 0.821612 | 0.00749 |
| RN7SKP23 | 0.076765 | 0.007137 | 0.825641 | 0.034171 |
| SAP30L-AS1 | 0.494734 | 0.273504 | 0.894912 | 0.019959 |
| SH3BP5-AS1 | 0.594481 | 0.446036 | 0.792331 | 0.000388 |
| SSBP3-AS1 | 0.466111 | 0.222434 | 0.976737 | 0.043143 |
| TMED2-DT | 0.217428 | 0.053387 | 0.885514 | 0.0332 |
| TSPOAP1-AS1 | 0.564932 | 0.421046 | 0.75799 | 0.00014 |
| Z97832.2 | 0.206748 | 0.051997 | 0.82206 | 0.025209 |
| Z97989.1 | 0.37456 | 0.152115 | 0.922296 | 0.032688 |
| AC004687.1 | 0.633219 | 0.501205 | 0.800004 | 0.000128 |
| AC010335.1 | 0.092184 | 0.00887 | 0.958065 | 0.045953 |
| AC020978.4 | 0.606449 | 0.391139 | 0.940281 | 0.025407 |
| AC093591.2 | 0.133866 | 0.02153 | 0.832322 | 0.03102 |
| AC098484.2 | 0.347377 | 0.182489 | 0.661247 | 0.001285 |
| AC099791.2 | 0.455763 | 0.221515 | 0.937724 | 0.032791 |
| AF127577.4 | 1.389749 | 1.021533 | 1.890688 | 0.036116 |
| AL035461.2 | 0.773238 | 0.62954 | 0.949736 | 0.014223 |
| AL109811.2 | 0.762328 | 0.633021 | 0.918049 | 0.004215 |
| AL117327.1 | 1.513043 | 1.052812 | 2.174461 | 0.025214 |
| AL162171.1 | 1.879179 | 1.163735 | 3.034464 | 0.009875 |
| AP003071.4 | 0.47149 | 0.273911 | 0.811587 | 0.00666 |
| DGCR11 | 1.444172 | 1.081047 | 1.929271 | 0.012869 |
| FAM198B-AS1 | 1.374884 | 1.048959 | 1.802079 | 0.021099 |
| LINC00941 | 1.162339 | 1.024571 | 1.318633 | 0.019436 |
| LINC01572 | 1.5915 | 1.056398 | 2.397649 | 0.026259 |
| LINC01871 | 0.870119 | 0.787569 | 0.961322 | 0.006227 |
| LINC02246 | 1.711124 | 1.170616 | 2.501201 | 0.005549 |
| PAPPA-AS1 | 1.258701 | 1.00693 | 1.573426 | 0.04332 |
| PCBP1-AS1 | 0.757232 | 0.631118 | 0.908548 | 0.002774 |
| AC006329.1 | 1.141097 | 1.008685 | 1.290891 | 0.035961 |
| AC012321.1 | 1.208947 | 1.006696 | 1.451833 | 0.042214 |
| AC015813.1 | 0.800684 | 0.645992 | 0.992421 | 0.042415 |
| AC022762.2 | 0.501559 | 0.266622 | 0.943513 | 0.032329 |
| AC084824.4 | 0.672764 | 0.474608 | 0.953652 | 0.025977 |
| AC092747.4 | 0.737678 | 0.59092 | 0.920884 | 0.007184 |
| LINC00852 | 0.46369 | 0.293779 | 0.731871 | 0.000965 |
| AC104041.1 | 1.376162 | 1.116508 | 1.696199 | 0.002762 |
| AL137802.2 | 1.333802 | 1.045896 | 1.70096 | 0.020251 |
| CASC19 | 1.145391 | 1.003876 | 1.306855 | 0.043648 |
| GATA2-AS1 | 1.188132 | 1.029467 | 1.371252 | 0.018421 |
| LINC01234 | 1.149778 | 1.047894 | 1.261569 | 0.003197 |
| UBAC2-AS1 | 1.592495 | 1.179331 | 2.150405 | 0.002395 |
| AC005076.1 | 1.328724 | 1.038286 | 1.700406 | 0.023913 |
| AC010894.2 | 1.283525 | 1.058067 | 1.557025 | 0.01132 |
| AC012640.2 | 1.327234 | 1.074488 | 1.639431 | 0.008626 |
| AC024060.2 | 0.763977 | 0.618876 | 0.943099 | 0.012242 |
| AC091153.3 | 1.385314 | 1.032869 | 1.858025 | 0.029566 |
| AC125616.1 | 2.452959 | 1.18181 | 5.091351 | 0.016026 |
| AL139289.2 | 1.300099 | 1.049368 | 1.610738 | 0.01636 |
| LINC01063 | 1.382011 | 1.110699 | 1.719598 | 0.003714 |
| LINC01106 | 1.328497 | 1.106648 | 1.59482 | 0.002311 |
| RNU6ATAC35P | 0.341208 | 0.117373 | 0.991907 | 0.048281 |
| USP2-AS1 | 1.666604 | 1.228496 | 2.260952 | 0.001029 |
| VPS9D1-AS1 | 1.185029 | 1.049434 | 1.338145 | 0.006178 |
| AC087854.1 | 0.209394 | 0.061186 | 0.716598 | 0.012744 |
| AC127521.1 | 0.498981 | 0.289445 | 0.860204 | 0.012353 |
| AL132989.1 | 0.268598 | 0.07298 | 0.988559 | 0.048011 |
| AC002553.2 | 0.659113 | 0.476058 | 0.912556 | 0.012032 |
| AC004908.2 | 0.66287 | 0.459015 | 0.957259 | 0.028313 |
| AC005674.2 | 0.482385 | 0.281631 | 0.826244 | 0.007928 |
| AC006480.2 | 0.339364 | 0.137809 | 0.835708 | 0.018759 |
| AC007610.4 | 0.033297 | 0.001133 | 0.978365 | 0.048536 |
| AC007686.3 | 0.10634 | 0.026965 | 0.419358 | 0.001368 |
| AC008764.8 | 0.443953 | 0.226578 | 0.869875 | 0.017973 |
| AC011498.6 | 0.609995 | 0.394984 | 0.942049 | 0.025801 |
| AC016773.1 | 0.607491 | 0.441155 | 0.836542 | 0.002263 |
| AC021321.1 | 0.476806 | 0.228542 | 0.994759 | 0.048385 |
| AC021739.2 | 0.339298 | 0.121141 | 0.950329 | 0.039694 |
| AC023830.3 | 0.10222 | 0.010662 | 0.979971 | 0.047984 |
| AC024361.3 | 0.506262 | 0.270302 | 0.948204 | 0.033497 |
| AC027682.7 | 0.10209 | 0.013142 | 0.79305 | 0.029136 |
| AC048341.1 | 0.374181 | 0.144543 | 0.968649 | 0.042806 |
| AC087289.2 | 0.373835 | 0.14033 | 0.995886 | 0.049044 |
| AC090772.1 | 0.358206 | 0.137508 | 0.933118 | 0.035582 |
| AC092375.2 | 0.258243 | 0.121398 | 0.549348 | 0.000439 |
| AC114730.3 | 0.457135 | 0.258144 | 0.809521 | 0.007259 |
| AC122688.3 | 0.623949 | 0.407915 | 0.954394 | 0.029613 |
| AC135050.6 | 0.801719 | 0.650536 | 0.988036 | 0.038185 |
| AL022328.3 | 0.615669 | 0.382001 | 0.992269 | 0.046389 |
| AL139287.1 | 0.734468 | 0.581779 | 0.927232 | 0.00945 |
| AL139349.1 | 0.388878 | 0.185335 | 0.815961 | 0.012495 |
| AL158163.1 | 0.352908 | 0.13773 | 0.904262 | 0.030038 |
| AL390066.1 | 0.309067 | 0.13342 | 0.715951 | 0.006152 |
| AL390719.2 | 0.832155 | 0.719406 | 0.962574 | 0.013381 |
| AL450384.2 | 0.534226 | 0.358856 | 0.795299 | 0.002014 |
| DCST1-AS1 | 0.737328 | 0.592619 | 0.917374 | 0.006265 |
| DPP9-AS1 | 0.112728 | 0.014793 | 0.859035 | 0.035152 |
| KTN1-AS1 | 1.27543 | 1.05757 | 1.53817 | 0.010907 |
| LINC01089 | 0.823093 | 0.703308 | 0.963279 | 0.015258 |
| SEMA6A-AS1 | 0.566522 | 0.365478 | 0.878158 | 0.011055 |
| Z99129.3 | 3.90E-05 | 3.79E-09 | 0.400571 | 0.031235 |
| ZSWIM8-AS1 | 0.641274 | 0.433318 | 0.949032 | 0.026315 |
| AC007952.6 | 0.00932 | 0.000919 | 0.094512 | 7.60E-05 |
| AC009949.1 | 0.001203 | 4.26E-06 | 0.339632 | 0.019542 |
| AC137894.1 | 0.415748 | 0.20927 | 0.825951 | 0.012213 |
| AL365361.1 | 0.540952 | 0.394789 | 0.74123 | 0.000132 |
| LINC02325 | 0.099539 | 0.020743 | 0.477651 | 0.003935 |
| MGAT3-AS1 | 0.407855 | 0.176997 | 0.939819 | 0.035231 |
| SIRPG-AS1 | 3.09E-05 | 6.77E-08 | 0.014104 | 0.000888 |
| AC012557.1 | 0.410028 | 0.193435 | 0.869146 | 0.020027 |
| AC027097.2 | 0.605726 | 0.378809 | 0.968573 | 0.036323 |
| AC090825.1 | 0.695502 | 0.501239 | 0.965056 | 0.029796 |
| AL122010.1 | 0.64217 | 0.420143 | 0.981529 | 0.040747 |
| AL158206.1 | 1.226776 | 1.011262 | 1.488218 | 0.038118 |
| AP001972.5 | 0.736408 | 0.608478 | 0.891234 | 0.001674 |
| AC068491.4 | 2.810274 | 1.24166 | 6.360549 | 0.013163 |
| AC087752.4 | 0.365493 | 0.213676 | 0.625176 | 0.000238 |
| AC098487.1 | 1.414761 | 1.132152 | 1.767914 | 0.002276 |
| AC115618.3 | 0.782829 | 0.637626 | 0.961098 | 0.019334 |
| AL035071.1 | 0.805481 | 0.656387 | 0.988441 | 0.038331 |
| AL512274.1 | 0.851293 | 0.749165 | 0.967342 | 0.013542 |
| FOXD1-AS1 | 1.155323 | 1.030811 | 1.294875 | 0.013082 |
| AC012181.1 | 0.631487 | 0.426609 | 0.934755 | 0.021611 |
| AC016747.4 | 0.231682 | 0.058426 | 0.918707 | 0.037471 |
| AC090241.3 | 0.023979 | 0.000592 | 0.971671 | 0.048248 |
| AC092720.1 | 0.37584 | 0.149374 | 0.945651 | 0.037648 |
| AC098869.2 | 0.107657 | 0.019638 | 0.590202 | 0.010248 |
| AC114980.1 | 0.108794 | 0.020594 | 0.574749 | 0.008999 |
| AL049597.2 | 0.671635 | 0.468562 | 0.96272 | 0.030252 |
| AL138921.2 | 0.311408 | 0.122267 | 0.793137 | 0.014452 |
| AP001767.4 | 0.242949 | 0.083343 | 0.708203 | 0.009541 |
| AP006621.4 | 0.425485 | 0.2173 | 0.83312 | 0.012684 |
| AC017116.1 | 0.819945 | 0.685543 | 0.980698 | 0.029755 |
| AC105749.1 | 0.3226 | 0.149635 | 0.6955 | 0.003897 |
| AC108134.1 | 0.560071 | 0.361143 | 0.868573 | 0.009616 |
| AC138956.1 | 0.336484 | 0.121244 | 0.933829 | 0.03649 |
| AC243960.1 | 0.641659 | 0.503439 | 0.817828 | 0.000337 |
| AL096816.1 | 0.10913 | 0.037189 | 0.320241 | 5.50E-05 |
| AL135818.1 | 0.55744 | 0.380784 | 0.81605 | 0.002653 |
| AL359091.5 | 1.214313 | 1.000787 | 1.473395 | 0.049075 |
| CHKB-DT | 1.321197 | 1.063268 | 1.641694 | 0.011951 |
| CHRM3-AS2 | 0.460866 | 0.305549 | 0.695132 | 0.000221 |
| CYTOR | 1.260394 | 1.086727 | 1.461815 | 0.002217 |
| LINC00426 | 0.612692 | 0.470383 | 0.798056 | 0.000281 |
| LINC00861 | 0.471082 | 0.323719 | 0.685526 | 8.40E-05 |
| LINC00996 | 0.403894 | 0.232489 | 0.701671 | 0.001294 |
| MIR3142HG | 0.507971 | 0.356605 | 0.723587 | 0.000175 |
| AC078777.1 | 0.499875 | 0.269606 | 0.926814 | 0.027719 |
| AC093330.2 | 0.000546 | 3.60E-07 | 0.828655 | 0.044404 |
| AL392089.1 | 1.266649 | 1.073728 | 1.494233 | 0.005051 |
